# Successful use of ofatumumab in two cases of early-onset juvenile SLE with thrombocytopenia caused by a mutation in protein kinase C *δ*

**DOI:** 10.1186/s12969-018-0278-1

**Published:** 2018-09-26

**Authors:** Linda Lei, Sabina Muhammad, Muthana Al-Obaidi, Neil Sebire, Iek Leng Cheng, Despina Eleftheriou, Paul Brogan

**Affiliations:** 1 UCL Medical School, London, UK; 2 Paediatric Rheumatology Department, Great Ormond Street Hospital for Children NHS Trust, London, UK; 3 Department of Paediatric Histopathology, Great Ormond Street Hospital, London, UK; 4 Infection, Inflammation and Rheumatology Section, Infection, Immunity, Inflammation and Physiological Medicine Programme, UCL Institute of Child Health, London, UK; 5 ARUK centre for adolescent rheumatology, London, UK

**Keywords:** Ofatumumab, Juvenile SLE, Rituximab, Protein kinase C *δ* deficiency, Acute tubulointerstitial nephritis

## Abstract

**Background:**

We previously described an endogamous Pakistani kindred in whom we identified a novel homozygous missense mutation in the *PRKCD* gene encoding for protein kinase C *δ* (PKC*δ*) as a cause of monogenic systemic lupus erythematosus (SLE). PKC*δ* has a role in the negative regulation of B cells. Given the nature of the disease, a logical targeted therapeutic approach in these patients is B cell depletion. Indeed, the 3 siblings all had a marked clinical response and resolution of symptoms with rituximab, although 2 of the siblings had severe reactions to rituximab thus precluding further treatment with this. We therefore describe the first successful use of ofatumumab for this rare form of monogenic SLE.

**Case presentation:**

All three affected siblings presented with SLE before the age of 3-years with lethargy, intermittent fever, thrombocytopenia, cutaneous involvement, alopecia, and hepatosplenomegaly. Tubulointerstitial nephritis was also present in 1 of the siblings. Homozygosity mapping followed by whole exome sequencing identified a homozygous missense mutation in *PRKCD* (p.Gly432Trp), subsequently confirmed by Sanger sequencing to be present in all 3 siblings. All 3 patients were initially treated with rituximab, however 2 of the siblings developed severe infusion-related reactions. For subsequent disease flare in these individuals we therefore used an alternative B cell depleting agent, ofatumumab (300 mg/1.73m^2^ on day 1; 700 mg/1.73m^2^ on day 15). This resulted in marked clinical improvement in both patients. To the best of our knowledge, this is the first report describing the successful use of ofatumumab for PKC*δ* deficiency.

**Conclusions:**

PKC*δ* deficiency causes a monogenic form of SLE which responds well to B cell depletion. Ofatumumab is also likely to have a therapeutic role for sporadic juvenile SLE (jSLE) patients intolerant of rituximab.

## Background

Juvenile SLE (jSLE) is rare before 5 years of age and can present with severe and atypical disease. There is an ever-expanding list of monogenic causes described, with a range of cutaneous and systemic features including cold-induced chilblain lupus, or mucocutaneous involvement, through to severe systemic and organ threatening manifestations such as renal involvement [1]. Awareness of monogenic forms of SLE is important, particularly for patients who present with SLE early in life. Moreover, there may be more targeted therapeutic options for such patients as compared to standard treatments for sporadic SLE [2]. The complexity in identifying a monogenic cause of lupus highlights the diagnostic importance of next generation sequencing (NGS) in this context, which can have major therapeutic and prognostic impact, in addition to facilitating family counselling. Indeed, the conventional approach of gene-by-gene testing by Sanger sequencing is increasingly superseded by NGS, particularly since there is a growing list of monogenic forms of SLE [2, 3].

**Fig. 1:**
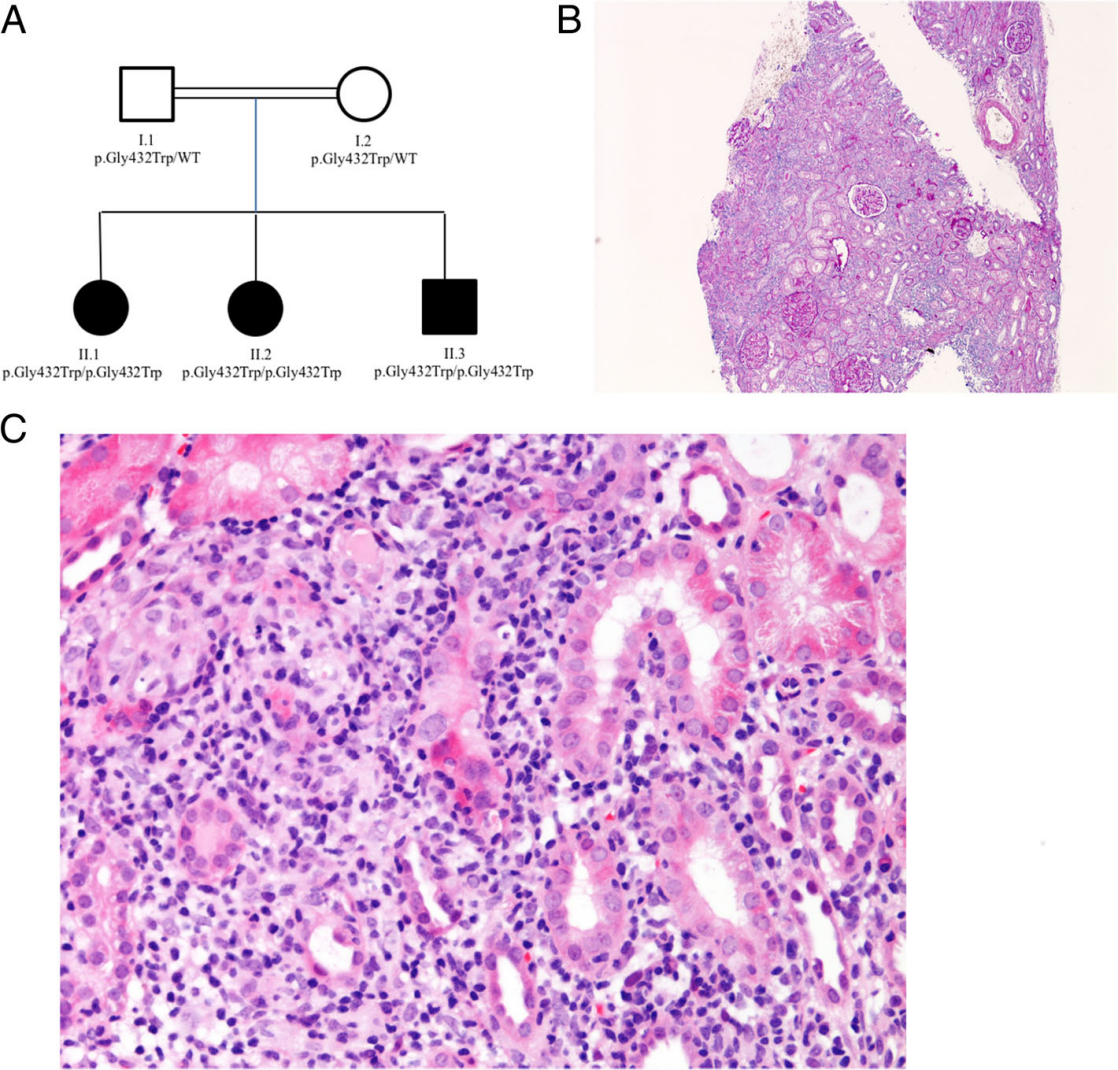
Pedigree of the family and renal histology. **a** The affected siblings (shaded) are homozygous for the *PRKCD* mutation as indicated and the parents are carriers of the mutation. WT, wild type. **b** Low power (Periodic acid–Schiff stain, original magnification × 40) and Fig. 1c higher power (Haematoxylin and Eosin stain, original magnification × 200) showing severe acute tubulointerstitial nephritis with marked predominantly mononuclear inflammatory infiltrate and associated mild acute tubular damage

We previously described an endogamous Pakistani kindred (Fig. 1a), with a homozygous missense mutation (p.Gly432Trp) in the *PRKCD* gene which codes for the active region of protein kinase C *δ* (PKC*δ*) as a cause of monogenic SLE [4]. *PRKCD* is located on chromosome 3p21.31, and its protein product PKC*δ* has a role in the negative regulation of B cells. Mice lacking in PKC*δ* have expansion and dysregulation of B cells [5], that might suggest a therapeutic role for B cell depletion in the human disease caused by *PRKCD* mutations. Herein we describe a novel disease manifestation of PKC*δ* deficiency, acute tubulointerstitial nephritis (TIN), and successful response to the B cell depleting agent ofatumumab in two of the siblings who developed hypersensitivity reactions to rituximab.

## Case presentation

The pedigree is shown in Fig. 1a. The parents were first cousins. The first two siblings (II-1 and II-2) became symptomatic at 12 months of age, and II-3 became symptomatic at 26 months of age. All three affected siblings (II-1, II-2, and II-3) presented with an SLE-like phenotype (Table 1) consisting of constitutional symptoms (intermittent fever, night sweats and fatigue), severe thrombocytopenia, cutaneous involvement, scarring alopecia, and hepatosplenomegaly. The cutaneous involvement in both siblings consisted of a photosensitive rash starting on the scalp and spreading to the malar area and nasal bridge with a petechial rash on the palms, soles, fingers and toes. II-1 also had ulcers on her palate on an oral examination. II-2 had dilated nailfold capillaries on the fifth finger of her left hand. II-1 had a palpable spleen measuring 2-3 cm with palpable cervical lymphadenopathy (anterior and posterior chains) and inguinal lymphadenopathy. II-2 presented with a palpable liver edge and a palpable spleen measuring 2-3 cm with cervical and inguinal lymphadenopathy. There was no arthritis in both siblings. There was no abnormality detected in the cardiac or central nervous system for either II-1 or II-2, and they were both afebrile. A summary of immunological and other relevant investigations for all 3 siblings is provided in Tables 2 and 3. Based on the clinical and pathological results, the siblings fulfilled the classification criteria for SLE (Table 1). Homozygosity mapping followed by whole exome sequencing identified a homozygous missense mutation (p.Gly432Trp) in *PRKCD* in II-1 and II-2, subsequently confirmed by Sanger sequencing to be present in all 3 siblings (Fig. 1a). Full details regarding the methodology for the various gene screening strategies are available elsewhere [4].

**Table 1:** Systemic Lupus International Collaborating Clinics (SLICC) criteria and the presence (√) or absence (x) for each affected sibling [25]

	II-1	II-2	II-3
Acute cutaneous lupus	√	√	√
Chronic cutaneous lupus	x	x	x
Oral or nasal ulcers	√	x	x
Non-scarring alopecia	√	√	√
Arthritis	x	x	x
Serositis	x	√	x
Renal	x	√	x
Neurologic	x	x	x
Haemolytic anaemia	√	√	√
Leukopenia	√	√	x
Thrombocytopenia	√	√	√
ANA (above lab range)	√	√	√
Anti-dsDNA(above lab range)	√	√	√
Anti-Sm	x	x	√
Anti-phospholipid antibody	√	√	x
Low complement	√	√	√
Direct Coombs’ test	not assessed	not assessed	not assessed

*WCC* White cell count, *ANA* Anti-nuclear antibodies, *ds-DNA* Double-stranded deoxyribonucleic acid, *Sm* Smith. To fulfil these classification criteria, 4 or more criteria must be present (including at least one clinical and one immunological feature), or biopsy proven lupus nephritis with positive ANA or anti-DNA antibodies

**Table 2:** Extractable nuclear antigen (ENA) antibody typing for siblings II-1 and II-2 at initial presentation, at relapse of disease and 8 months after ofatumumab

ENA antibody typing	Patient II-1			Patient II-2		
	At presentation	At relapse	8 months after ofatumumab	At presentation	At relapse	8 months after ofatumumab
anti-NRNP	negative	negative	positive	negative	negative	equivocal
anti-SME	negative	negative	positive	negative	negative	positive
anti-Ro	negative	negative	positive	negative	negative	negative
anti-La	negative	negative	negative	negative	negative	negative
anti-Jo-1	negative	negative	negative	negative	negative	negative
anti-Scl70	negative	negative	negative	negative	negative	negative
other ENA antibodies	weakly positive	negative	negative	weakly positive	negative	negative

**Table 3:** Laboratory data of siblings II-1 and II-2 at initial presentation, at relapse of disease requiring ofatumumab infusion and at follow-up 8 months after ofatumumab infusion

Investigations	Patient II-1 (reference range)			Patient II-2 (reference range)		
	At presentation	At relapse	8 months after ofatumumab	At presentation	At relapse	8 months after ofatumumab
FBC × 10^12/L (RR 4.00–5.20)	3.88	3.92	4.76	3.6	3.01	5.26
Hb g/L (RR 115–145)	96	102	118	97	73	128
Platelets × 10^9/L (RR 150–450)	85	5	209	13	63	397
WCC × 10^9/L (RR 0.0–10.0)	5.94	6.54	6.58	9.60	2.78	7.83
Lymphocytes × 10^9/L (1.5–7.0)	1.79	1.69	1.65	1.70	0.48	1.86
Neutrophils × 10^9/L (RR 1.5–8.0)	2.17	4.20	1.83	1.01	2.23	4.90
CD 19 lymphocyte subset %	21.0	not assessed	0.0	25.0	23.7	0.5
CD 3 lymphocyte subset %	73.0	not assessed	97.5	65.0	83.0	96.0
CD 16+ CD 56+ lymphocyte subset %	4.0	not assessed	1.7	8.0	5.0	0.7
CD 3+ CD 56+ lymphocyte subset %	0.2	not assessed	not assessed	0.2	0.8	not assessed
CD 3+ CD4+ lymphocyte subset %	46.0	not assessed	49.7	32.0	38.0	41.5
CD 3+ CD 8+ lymphocyte subset %	21.0	not assessed	40.6	37.0	37.0	42.2
ESR mm/hr. (RR 0–10)	128	132	50	130	75	1
CRP mg/L (RR < 20)	21	19	< 5	28	21	< 5
Urea	3.4	3.9	3.4	4.8	7.8	4.1
Creatinine umol/L (RR 28–57)	36	25	40	33	83	52
Urine creatinine mmol/L	3.4	1.5	3.5	2.8	1.1	0.5
Urine albumin/creatinine ratio mg/mmol (RR 0.2–4.5)	1.5	5.3	3.1	8.9	1.1	not assessed
Urine NAG/creatinine ratio units/mmol (RR 2–20)	not assessed	not assessed	4	not assessed	67	74
Urine retinol binding protein ug/L	not assessed	not assessed	39	not assessed	not assessed	161
Urine retinol/creatinine ratio ug/mmol (RR 5–41)	not assessed	not assessed	11	not assessed	not assessed	85
ANA	1:1280	not assessed	1:640	Negative	not assessed	not assessed
Anti-DNA IU/ml (RR 0–9.9)	65	66	16	43	16	4.8
IgG G/L (RR 5.4–16.1)	16.8	22.3	7.66	21.9	18.5	7.12
IgA G/L (RR 0.5–2.4)	2.19	2.35	1.15	2.69	3.34	1.72
IgM G/L (RR 0.5–1.8)	1.63	1.31	0.35	2.90	2.36	0.86
C3 G/L (RR 0.75–1.65)	0.83	0.41	1.51	0.64	0.91	1.37
C4 G/L (RR 0.14–0.54)	0.10	0.03	0.19	0.08	0.15	0.29

*RR* Reference range, *FBC* Full blood count, *Hb* haemoglobin, *WCC* White cell count, *ESR* Erythrocyte sedimentation rate, *CRP* C reactive protein, *NAG* Nitrosaminoglycan, *DNA* deoxyribonucleic acid

In addition to the investigations included in Tables 1, 2 and 3, additional autoimmune workup in II-1 at the time of initial presentation revealed: IgA 2.20 G/L (RR 0.4–2.0), tissue transglutaminase (TTG) IgA screening negative, C1q 18.0 U/ml (RR 0–15), C1 esterase inhibitor 438.0 mg/L (RR 150–350), C1 esterase functional assay > 93% (RR > 84%), C1q level 53.0 mg/L (RR 50–250), proteinase 3 antineutrophil cytoplasmic antibodies (PR3-ANCA) 0.40 IU/ml (RR 0–1.99), myeloperoxidase (MPO) ANCA 0.40 IU/ml (RR 0–3.49), rheumatoid factor < 0.20 IU/ml (RR 0. 20), thyroid peroxidase antibodies < 33.4 IU/ml (RR < 60), classical and alternative functional complement assays were normal at 110% (RR > 40) and 92% (RR > 10) respectively.

Full autoimmune workup (see also Tables 1, 2 and 3) for II-2 revealed: IgA 2.88 G/L (RR 0.4–2.0), TTG IgA screening negative, C1q 9.0 U/ml (RR 0–15), C1 esterase inhibitor 434.0 mg/L (RR 150–350), C1 esterase functional assay > 93% (RR > 84%), C1q level 42.0 mg/L (RR 50–250), PR3-ANCA 0.60 IU/ml (RR 0–1.99), MPO-ANCA 0.80 IU/ml (RR 0–3.49), rheumatoid factor < 0.20 IU/ml (RR 0. 20), thyroid peroxidase antibodies < 33.4 IU/ml (RR < 60), and normal classical and alternative complement functional pathway assay at 75% (RR > 40) and 99% (RR > 10) respectively.

ENA antibody typing was also done for II-1 and II-2 at presentation, at time of relapse and at 8 months follow-up after ofatumumab (Table 2). ASMA and anti-LKM autoantibodies were not assessed.

II-1 and II-2 failed initial treatment with high dose corticosteroids, hydroxychloroquine 5 mg/kg per day and azathioprine 2 mg/kg per day. II-2 developed spontaneous gingival bleeding, and platelet-count 4 ×  10^9/L (reference range [RR]150–450). Following IVIG 2 g/kg, platelets rose transiently to 30 × 10^9/L. Consequently, she received rituximab (750 mg/m^2^ (maximum 1 g) on day 1 and 15). The platelet count rapidly normalised (179 × 10^^^9/L), with near-normalization of other laboratory parameters [6]. However, during the second rituximab infusion she developed urticaria, wheeze, and hypotension despite premedication, necessitating discontinuation of the infusion discontinuation, and treatment with hydrocortisone and volume expansion with normal saline. Despite that, she had a good therapeutic response and adequate B cell depletion (peripheral blood CD19 count less than 1%).

Twelve months later, II-2 was re-admitted with lethargy, poor appetite, night sweats and was found to have renal impairment, ongoing acute phase response, and return of B cells (CD19 23.7%): blood pressure 102/43 mm/Hg; urea 7.8 mmol/L (RR 2.5–6.0); creatinine 83 μmol/L (RR 30–48); and ESR 75 mm/hr. (RR 0.0–10.0). Urinary chemistry was consistent with tubulopathy: urine (_u-_) sodium 122 mmol/l, _u-_potassium 36 mmol/L, _u-_pH 7.0, _u-_nitrosaminoglycan [NAG]/creatinine ratio 67 (RR 2–20), _u-_albumin/creatinine ratio 1.1 mg/mmol (RR 0.2–4.5). Renal biopsy confirmed acute tubulointerstitial nephritis, with coexistent chronic changes (Fig. 1b, and c). She received IV methylprednisolone (30 mg/kg) for 3 days, thereafter continuing on a tapering course of prednisolone 40 mg/day. In view of her genotype and excellent previous therapeutic response (but severe drug reaction) to rituximab, ofatumumab (300 mg/1.73m^2^ on day 1; 700 mg/1.73m^2^ on day 15) was commenced. Again, B cells depleted successfully (CD19: 0.1%), and her lethargy, poor appetite, and night sweats had resolved completely two weeks later. At follow-up eight months later, her renal function had improved (urea 4.1 mmol/L; creatinine 52 μmol/L) allowing successful prednisolone taper; but tubular function was not completely normal (_u-_NAG:creatinine ratio 74), indicating that the chronic changes identified on renal biopsy may be irreversible, and not amenable to improvement with immunosuppressive treatment. At follow-up eight months after ofatumumab infusion, B cells were still only gradually recovering (CD19 0.5%) (Table 3). In terms of the antibodies, anti-dsDNA antibodies was 16 IU/ml (RR 0–9.9) at the time of relapse and this dropped to 4.8 IU/ml (RR 0–9.9) eight months after ofatumumab infusion (Table 3). Hb recovered from 73 g/L (RR 115–145) to 128 g/L (RR 115–145), and the white cell count also improved from 2.78 × 10^9/L (RR 5.0–15.0) to 7.83 × 10^9/L (RR 5.0–15.0) (Table 3). At follow-up eight months after ofatumumab infusion, there are no adverse events reported.

A similar story gradually evolved in II-1. Although the symptoms (thrombocytopenia, rash, and alopecia) initially responded to a first course of rituximab (2 infusions of 750 mg/m^2^, on day 1 and 15), return of these symptoms, plus increasing lethargy and night sweats within a year indicated the need for retreatment. During the second of two planned rituximab infusions, she developed severe diffuse cutaneous erythema and tachycardia necessitating infusion discontinuation, although again she still adequately B cell depleted and had symptomatic relief for 32 months. She then re-presented with platelets of 5 × 10^9^/L, and thus received ofatumumab (same dose as for patient II-2) without incident. Her platelet count rapidly rose to 209 × 10^9^/L, with sustained therapeutic response and B cell depletion (CD19 0.0%) now 8 months later. At follow-up, the cutaneous symptoms had resolved in both II-1 and II-2 although the scarring alopecia is more likely to be chronic. The hepatosplenomegaly, lethargy and night sweats had also resolved. II -2 has kidney involvement that is likely to be chronic. In terms of the serological response, anti-dsDNA antibodies was 66 IU/ml (RR 0–9.9) at the time of relapse and this dropped to 16 IU/ml (RR 0–9.9) eight months after ofatumumab infusion (Table 3). Hb improved from 102 g/L (RR 115–145) to 118 g/L (RR 115–145) (Table 3). At follow-up eight months after ofatumumab infusion, there are no adverse events reported.

II-3 developed symptoms at age 26 months: facial rash, alopecia, and platelets 6 × 10^9^/L. He required IV methylprednisolone, IVIG 2 g/Kg, and two infusions of rituximab which he received without incident and with sustained efficacy at last follow-up despite return of B cells (CD19 0.1% post rituximab; 9.4%) 8 months after receiving rituximab.

## Discussion and conclusions

This report emphasises a number of novel observations: firstly, severe recalcitrant thrombocytopenia is a prominent feature of PKC*δ* deficiency; secondly, we provide the first description of acute TIN associated with PKC*δ* deficiency; thirdly, PKC*δ* deficiency is exquisitely sensitive to B cell depletion (with no obvious difference in efficacy between rituximab or Ofatumumab, notwithstanding the issues of hypersensitivity); and lastly, 2 of the siblings suffered intolerable infusion reactions to rituximab, but have so far responded well to ofatumumab, which may offer a less immunogenic alternative for patients with prior rituximab hypersensitivity.

PKC*δ* has been ascribed critical roles in the regulation of cellular proliferation and apoptosis, with important pro-apoptotic activity due to the inhibition of cell cycle progression [7]. PKC*δ*-driven apoptosis has also been reported in association with various DNA damaging agents, such as UV radiation and etoposide [6, 8, 9]. PKC*δ* regulates B cell negative selection, and deficiency of PKC*δ* causes systemic autoimmunity in mice [10], and also in humans with phenotypes that include lymphoproliferative disease and lupus-like autoimmunity [4, 11]. Belot et al. described 3 siblings from a consanguineous kindred with an increased number of immature B cells and a developmental shift towards naïve B cells with an immature phenotype, indicating that PKC*δ* is vital in regulating B cell maturation and tolerance [11], (and see Table 4). These observations almost certainly explain why autoimmunity in humans caused by PKC*δ* deficiency seems particularly amenable to treatments that target B lymphocytes, as illustrated by the kindred described herein.

**Table 4:** The phenotype and treatment of the 9 patients with PKC*δ* deficiency described to date, including the 3 siblings in this report

Mutation	Number of cases	Phenotype	Treatment	Outcomes	Reference number
c.1840C > T, p.R614W (homozygous)	1	SLE –like with lymphoproliferation	Corticosteroids and rapamycin	Reduction in size of lymph nodes and resolution of hepatosplenomegaly, transaminitis and ongoing acute phase response	[26]
G510S/G510S	3	SLE-like, lymphoproliferation and recurrent infections	Corticosteroids in all; Various DMARDS in 2 Rituximab in 1	Relapsing course in 2 cases; death age 13 in 1 case (septic shock)	[11, 27]
c.1352 + 1G > A	1	SLE-like and recurrent infections	Corticosteroids; Various DMARDs; rituximab; immunoglobin replacement	Good response	[28]
c.742G > A, p.Gly248Ser	1	SLE-like, CMV infection	Oral hydroxychloroquine; antibiotic and IVIG prophylaxis	Good response	[29]
c.1294G > T; p.Gly432Trp (homozygous)	3	SLE-like (see main text); severe haematological involvement in all 3; acute tubulointerstitial nephritis in 1	high dose corticosteroids in all 3; various DMARDS in 2; rituximab in 3; ofatumumab in 2	Good response to rituximab in all 3, but severe infusion reactions in 2 cases. Good response to ofatumumab in 2 cases	[4], and this report

*SLE* Systemic lupus erythematosus, *DMARDs* Disease modifying anti-rheumatic drugs, *CMV* Cytomegalovirus, *IVIG* Intravenous immunoglobin

Ofatumumab is a fully human monoclonal antibody targeting B cells that may provide an attractive alternative to rituximab since it can deplete rituximab-resistant cells that express low levels of CD20, possibly through targeting a different epitope of CD20 [12], or by increased complement-dependent cytotoxicity [13]. The first ofatumumab clinical trial in 2008 was in patients with refractory or relapsed chronic lymphocytic leukaemia, leading to Federal Drug Agency approval for this indication in 2009. Ofatumumab has since been used for various conditions including nephrotic syndrome, refractory follicular lymphoma, and multiple sclerosis [14–16], amongst others. Several trials have highlighted a potential role for ofatumumab in diseases that are refractory to conventional treatment. For example, in biological-naïve rheumatoid arthritis with an inadequate response to methotrexate, patients on ofatumumab had significantly improved clinical outcomes without detectable immunogenicity [17]. In comparison to rituximab, which is a chimeric antibody, ofatumumab is a fully humanised monoclonal antibody (generated via human immunoglobulin transgenic mice) which may explain this reduced immunogenicity, and perhaps why this was better tolerated than rituximab in our two patients.

Although rituximab did not demonstrate efficacy in clinical trials of patients with SLE (probably due to trial methodological considerations), there is an increasingly compelling real-world evidence base for the efficacy of B cell depletion with rituximab in patients with refractory SLE [18]. That said, severe infusion reactions are commonly reported and therefore alternative B cell depleting agents may be required. The first report of ofatumumab in SLE was in an adult in 2014 [19]; in addition, there has been a single report of its use in jSLE, where it was used successfully to treat a 20 year old with recalcitrant life-threatening autoimmune haemolytic anaemia (AIHA) [20]. This patient had also failed multiple drugs, and had also experienced a severe infusion reaction to rituximab [20]. The dose we used (300 mg/1.73m^2^ on day 1; 700 mg/1.73m^2^on day 15) was the same low-dose protocol as described by Bonnani [21] for nephrotic syndrome, but lower than that previously used in other reports for idiopathic nephrotic syndrome [22]. Both II-1 and II-2 have had an improvement in their symptoms following ofatumumab, including improvement (but not normalisation) of renal function in II-2.

The main limitations of our observations thus far are limited time to follow-up; and lack of detailed B cell immunophenotyping pre and post B cell depletion, since samples were not available to us for these studies. We anticipate an ongoing relapsing disease course with return of B cells. Therapeutic options beyond B cell depletion are extremely limited, although there could be a role for other B cell-targeting biologics. Ultimately, however, allogeneic haematopoietic stem cell transplantation may be required, although this has never been reported for PKC*δ* deficiency (Table 4). Autologous HSCT, although potentially less toxic than allogeneic and emerging as a possible treatment for severe SLE [23, 24], is unlikely to be a useful therapeutic option for this monogenic form of SLE.

In conclusion, we describe the first favourable report of the use of ofatumumab for the treatment of monogenic lupus caused by PKC*δ* deficiency. Ofatumumab could also have a role for sporadic jSLE patients intolerant of rituximab.

## Abbreviations

jSLE: juvenile SLE
PKC*δ*: Protein kinase C *δ*
SLE: Systemic lupus erythematosus
